# Concepts in soft-tissue reconstruction of the contracted hand and upper extremity after burn injury

**DOI:** 10.3389/fsurg.2023.1118810

**Published:** 2023-05-03

**Authors:** Colin T. McNamara, Matthew L. Iorio, Mark Greyson

**Affiliations:** Division of Plastic and Reconstructive Surgery, University of Colorado Anschutz Medical Center, Aurora, CO, United Sates

**Keywords:** burn, contracture, reconstruction, hand, upper extremity

## Abstract

Burns and their subsequent contracture result in devastating functional and aesthetic consequences which disproportionally affect the upper extremity. By focusing on reconstruction with analogous tissue and utilizing the reconstructive elevator, function can be restored concomitantly with form and aesthetic appearance. General concepts for soft-tissue reconstruction after burn contracture are presented for different sub-units and joints.

## 1. Introduction

Burns represent a significant burden to both the United States and international healthcare systems. Hand injuries represent a disproportionate number of these injuries; the hand represents between 3% and 5% of total body surface area but has been cited as being involved in between 40% and 70% of all burns (1–3). Across the globe more than 18 million people suffer from the sequelae of hand burns (4).

Contracture, stiffness and joint immobility are all secondary consequences of burn injury that can represent significant functional impairment. Intrinsically related to depth and size of burn (5), access to medical care (6) and post-burn care treatment, contracture can affect more than a third of burn patients even after treatment at a major burn center (1, 7, 8). The hand is the most common site for contractures, constituting up to 72% of all contractures (5, 8). Between 20% and 50% of those with hand burns will demonstrate diminished function and 5% may not be able to perform the activities of daily living (9, 10). This can represent long-term disability and impair the ability to work (11, 12).

In addition to functional defects, burns also have a significant aesthetic and psychologic component. Bodily location of burns as well as visible scarring has been shown to have significant impact on psychological outcomes—resulting in increased anxiety, depression and body image issues (13). Though regaining function has been a frequent target of literature, restoring the natural look, contour and appearance of the hand may be just as important. Articles in the plastic surgery literature have long noted the improvement in quality of life after aesthetic surgeries (14–16) and the effect of scars on quality of life after treatment (17). Recently, the effect of aesthetic restoration in hand surgery has also been raised (18, 19). For patients with rheumatoid arthritis, appearance has been identified as being both a primary motivator for surgical intervention (20) as well as independently correlated with post-operative satisfaction. In these patients, aesthetics was weighted just as heavily as improved function (18, 21, 22). In an attempt to guide both functional and aesthetic reconstruction of the hand, Rehim et al. described the functional aesthetic unit of the hand and established the principles of restoring the anatomic and topographic contours in order to reproduce the natural highlights of the hand (23).

Burn scar injuries result in devastating functional, psychological and aesthetic consequences for affected patients. Here we propose our algorithm for treatment of post-burn soft tissue contracture with a focus on restoring both function and appearance.

## 2. Principles

Functionally the goal with upper extremity burn scar reconstruction is to restore task-oriented motions. Restoration of pinch, grasp and power grip are the priority via soft tissue contracture release, joint contracture release and restoration of soft tissue pliability. Aesthetic reconstruction incorporates soft tissue pliability and adds in the concept of replacing “like with like” by shaping and excising tissue to re-establish the contours that reflect that natural features and shape of the hand.

Reconstruction of a contracture begins with a thorough history and physical exam. A history should include the information around the burn including the cause (thermal, electrical, chemical), time since initial injury, interventions performed as well as information regarding the patient including hand dominance, profession, hobbies, areas the patient feels functionally limited in as well as areas that concern the patient psychologically. Examination should include the skin contractures that are present, quality of the surrounding skin, the underlying joint and possibility for underlying capsular contracture, the motion of all possibly affected joints, sensation and vascular status. Additional work-up may include radiographs to assess for heterotopic ossification or bony ankylosis and possible angiography if considering major regional or free flap reconstruction. The most critical part of the initial consulatation however is establishing patient goals and understanding what their functional and psychological concerns are for reconstruction.

For the severely burned patient we attempt to work proximal to distal, ensuring larger range of motion prior to establishing finer motions. Additionally, as each joint has specific needs and functional goals, each area should be addressed individually. When performing joints concomitantly, recurrence and complications rise, with Balumuka et al. showing significantly higher recurrence when the elbow and shoulder were operated on concomittantly (24).

In general, we begin with release of the superficial contracture transversely across the area of maximal stricture and in line with the joint requiring release. Release should extend from healthy tissue through the whole scar, both in terms of depth and width. Others have recommended a fishmouth incision that extends into normal skin in order to ensure complete release (25, 26). In either method, the deeper structures and joint can be evaluated and release of the capsule or surrounding structures performed if necessary. A common error is incomplete release resulting in operations with low marginal benefit for the patient.

The focus then turns to reconstruction. The choice of soft tissue reconstruction is predicated on the depth of injury, exposure of underlying structures following release of scar contracture, and the availability of healthy surrounding tissue. Options for reconstruction include split and full thickness skin grafts, local tissue re-arrangement, local flaps, regional flaps, distant pedicled flaps such as the groin flap, and free tissue transfer. Although traditionally the reconstructive ladder has encouraged a step-wise approach, restoring a more natural and functional appearance may require jumping steps.

Split and full thickness skin grafts have long been the mainstay of both initial and secondary burn surgeries. Split-thickness grafts represent an easily performed, accessible surgery which can cover large areas of superficial tissue with relatively low donor morbidity. Disadvantages include high secondary contracture rates, poor aesthetics due to the interstices if meshed and the tendency to either hyper or hypopigment compared to surrounding tissue, especially in darker-skinned patients (27). Though it is often recommended to increase the thickness of the graft due to the inverse relationship with contracture (28), no difference has been shown between thin (0.015in) and thick (0.025in) split-thickness grafts used for the hand in terms of range of motion or appearance (29). Split-thickness grafts may also be slightly less durable and at higher risk of injury than full-thickness or flap based alternatives (27, 30). Full thickness grafts must be carefully de-fatted and carefully fixed. Immobilization can be helpful to aid graft take and reduce shear forces.

Dermal substitutes like Integra (Integra LifeSciences, Plainsboro, New Jersey) which is a bilayer, acellular, dermal regeneration matrix, can also be used to establish a thicker granulation bed for skin graft take and allow better tendon gliding (31). Combined with split-thickness grafting it showed no difference in elasticity compared with unburned skin (32). Dermal regeneration matrixes may help with re-contracture rates (33, 34) though the hand in particular has been noted to have a higher rate of re-contracture than other sites (35). The potential risks include infection-related loss, the cost of the product, as well as the need for a second operation given the three to four weeks of maturation it requires. However, this could be considered against the risk of initial skin graft loss or infection due to inadequate wound bed preparation.

**Figure 1:**
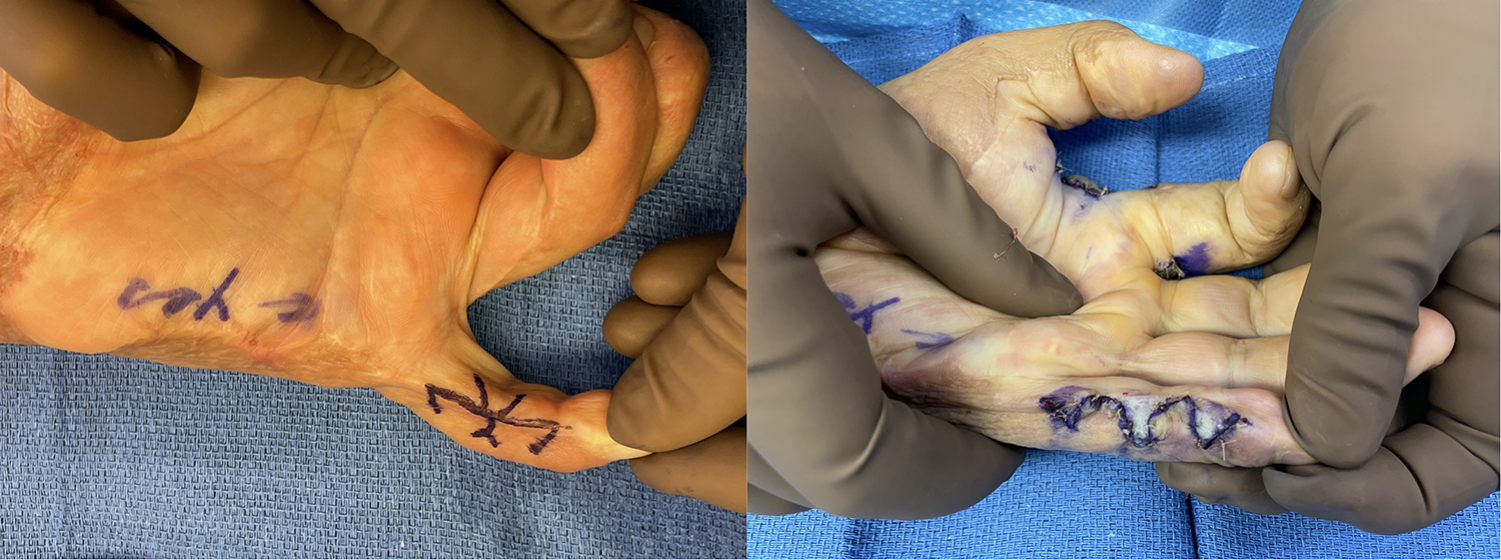
32yo M with 27% TBSA flame burns treated with STSG. He developed 20deg flexion contracture of his left small finger proximal interphalangeal joint and underwent a double opposing z-plasty with V-Y advancement (jumping man) flap for release in which he was able to achieve full extension.

Local tissue re-arrangements such as z-plasty are excellent options for linear bands that are surrounded by healthy tissue (Figure 1). They are powerful tools to restore normal contour, particularly when used as an adjunct to skin grafting and flap reconstruction. However, they must be used judiciously as a primary reconstructive tool, as they are most useful in burn scar areas which are adjacent to healthy, uninjured skin. It must be remembered that burn tissue is relatively inelastic and therefore may not reach the levels of lengthening as described for other scar releases when working entirely within a field of burn scar contracture. Concern for these local rotations, especially when rotating scarred tissue or areas of epifascial necrectomy, revolves around the lack of perfusion of this tissue which is compounded by the undermining often required that can leave these flaps ischemic (36).

When significant underlying structures including bone, tendon, nerve, or vessels become exposed; or if there is a need to re-establish a smooth, gliding surface, well vascularized tissue becomes a requirement. This can be performed using either local pedicled or free tissue transfer.

Local or regional pedicled options are advantageous as they simplify the operation into one location and do not require microsurgical expertise. The disadvantage is that for high energy trauma the flap often remains within the zone of injury or surrounding injuries may make these flaps infeasible. The majority of options are fasciocutaneous flaps that can provide relatively thin and durable tissue while remaining favorable for re-elevation during additional reconstructive procedures.

Choosing a local or regional pedicled flap will be dependent on the area of required coverage but can include the radial forearm flap, the posterior interosseous artery flap, and the lateral arm flap. The radial forearm flap is a work-horse flap and can be utilized for reconstruction of the forearm and elbow, and to the hand if harvested as a reverse flow flap. As both an antegrade and retrograde flap, the design can be based off of perforators, eliminating the need to sacrifice the radial artery while still providing coverage for the hand assuming (37, 38). Complications can include superficial radial nerve branch injury, cold intolerance, poor aesthetic donor site due to the need to skin graft, ischemic complications from artery ligation and functional limitations (39).

The lateral arm flap is a versatile flap that can also be used antegrade for arm coverage or retrograde in order to facilitate coverage of elbow wounds with the advantage that it can be closed primarily (40). The reverse posterior interosseous flap can also be designed is a flap which also does not sacrifice a major vessel and can be used to reach to the thumb or first webspace. However intact flow through the anterior interosseous artery must be present, and venous congestion is a known complication (41).

Pedicled groin or abdominal flaps represent other consistent, pedicled choices that do not require microsurgery, with the significant caveat of requiring adhesion/immobilization of the arm to the abdomen for 3–4 weeks and a second procedure for division. Thought should be given to location of injury and placement of the hand on the belly in terms of comfort and range of motion. Flaps can be based off of the deep or superficial inferior epigastric arteries (DIEA/SIEA), the superficial circumflex iliac artery (SCIA), superficial external pudendal artery (SEPA) or a combination of these for large or volar and dorsal defects (42). The groin flap is based on the SCIA, two finger breadths (2.5 cm) below the inguinal ligament and can cover defects 10 × 25 cm (43–45) with the lateral portion able to be significantly thinned (46). For the dorsum of the hand this also provides a natural position for the remainder of the arm, similar to a “hand in pocket.” The paraumbilical artery perforator flap is based off of the paraumbilical artery perforators from the deep inferior epigastric artery (47). These vessels can be identified via ultrasound or IcG angiography and then the flap raised 6–8 cm wide to the level of the anterior axillary line (48). With any pedicled flap, the safety of division also depends on the size of the defect. A large, well vascularized defect will have robust vascular ingrowth (42) and smaller defects may have higher failure rate (49). The pedicle can also be cross-clamped either intermittently or gradually in order to produce ischemia and speed up angiogenesis for secondary division (42).

Finally, free flaps represent the top of the reconstructive ladder, though with advances in microsurgical techniques and dissection, this concept has been turned into the reconstructive elevator (50). Using a free flap can help minimize donor morbidity by choosing areas which are less visible and can be closed primarily as well as by being able to choose the most thin, pliable yet hearty flaps from all over the body. Additionally, they may allow a quicker return to motion and activity using only one procedure. However though thin, pliable flaps can be found, they likely still represent tissue of slightly different quality, texture and coloration and this must be considered.

Typical flaps that are utilized are the radial forearm flap, anterolateral thigh flap (ALT), lateral arm flap, superficial circumflex iliac artery perforator flap (SCIP), medial sural artery perforator flap (MSAP), and the parascapular flap. Muscles that can be used as flaps are the gracilis and the latissimus muscles.

For all reconstructive options, post-operative therapy is a necessity in maintaining the gains that have been made in quality of the tissue and range of motion. Initial splinting followed by scar compression, massage and early mobilization are all critical portions of the after-care required.

## 3. Axilla

Contractures of the axillae can cause restriction in multiple planes of motion. Though full range is not required for activities of daily living, having at least 130 degrees of abduction and 140 degrees of flexion are required for basic tasks. In absence of this range, patients with axillary contractures alter the mechanics of their other joints in order to perform daily tasks (51, 52). It represents an area of particular difficulty due to its irregular contour. A variety of classification schemas based on this anatomy have been put forth which rate axillae contracture based on anterior, posterior or full cupola involvement (53–55). We find it more relevant for surgical planning to examine first the degree of initial contracture. As the axillae recruits nearly 16 cm of regional tissue to reach full abduction (56), severe contracture release can result in a large soft tissue deficit.

For more simple linear or edge contractures that have adjacent, unburned tissue, this same concept must be remembered and applied. Numerous local options have been described including z-plasty in series or parallel, v-y advancement, multiple v-y (m-plasty) (57), running y-v (58), trapeze-plasty (59), and square flap (60). Each of these involves multiple flaps in order to recruit enough tissue to restore sufficient soft tissue for full abduction. Often these must be combined with grafting in order to cover the defect as well as extend the distance between contracting bands. However, care must be taken when considering grafting these due to the complicated contour and higher risk for graft loss (61).

Local pedicled options include the parascapular flap (62), the posterior arm fasciocutaneous flap based off of a perforator from the profundal brachii (63), the thoracodorsal artery perforator flap (64), and the latissimus fasciocutaneous flap (65, 66). Muscle flaps, although proposed, are frequently too bulky for this area.

If no local options are available we again prefer thin fasciocutaneous free flaps such as the anterolateral thigh flap or medial sural artery perforator flap. These can be anastomosed to the thoracodorsal artery. Depending on the BMI of the patient and constriction of surrounding skin, these may need to undergo subsequent debulking. Chen et al. (67) describes ten ALT flaps for the axillae for which none required debulking however their population typically has low BMI and may not be representative of all populations.

Post-operatively an abduction splint of preferably over 100 degrees to allow the greatest functional recovery is applied. This is especially important in grafted wounds to reduce post-operative contracture.

## 4. Elbow

The elbow is one of the most often contracted joints after burns. It is typically held in flexion as a position of comfort. This can create extension limitations due to contracture in the antecubital fossa and relative strength of the flexors vs. extensors. Additionally the elbow is a common place for heterotopic ossification which can create a hard stop limiting both flexion and extension (8). Near full range of motion in the elbow is required for completion of normal activities of daily living (51). Without adequate elbow range of motion, a patient is unable to position the wrist and hand. Elbow contracture can be classified based on type of scar pattern or amount of extension. Both of these are relevant and play into the reconstructive decision-making.

Simple corded or linear scar patterns are the most straight-forward to reconstruct. These can be released with multiple z-plasties, or if soft, supple tissue is present to either side, V-Y or local advancement flaps such as the trapezoid advancement (68) can be utilized. If a significant fold of tissue exists, this can also be recruited to help increase extension (69).

It has been shown that the elbow recruits a significant amount of skin to go from fully flexed to fully extended—typically about 11 cm (56). This often results in a large soft tissue deficit when tight contractures are released. Split-thickness grafting alone for these defects presents a three-fold higher risk of re-contracture (70) and bringing in vascularized tissue represents the best opportunity to maintain the reconstruction in the setting of large extension deficits. If contracture has been prolonged, this may need to be combined with additional procedures such as biceps lengthening, myotomy and/or joint capsule release (71).

**Figure 2:**
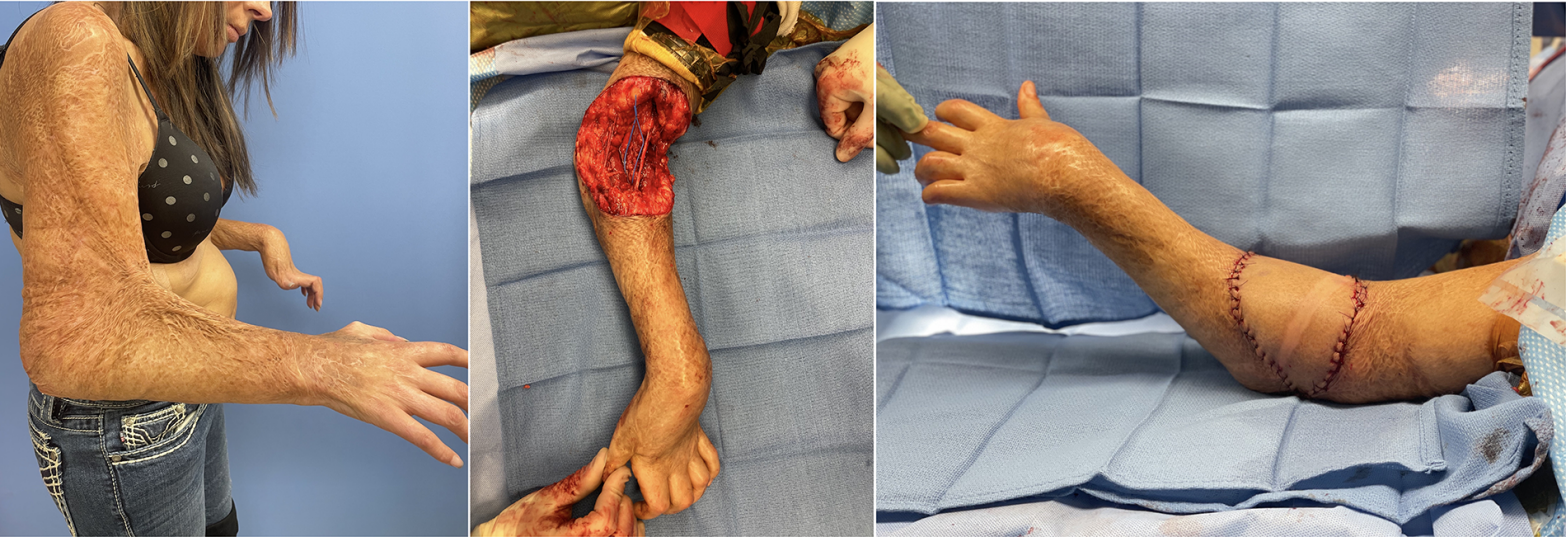
32 F with 40% TBSA flame burns including her bilateral arms initially treated with STSG. Developed 90deg rigid flexion contracture of her elbows. Her skin and lacertus fascia were released with lengthening of her biceps tendon. A 12 × 12 cm soft tissue defect was repaired with free ALT to her recurrent radial vessel. After repair was able to extend to 30deg.

Local pedicled options include the lateral arm flap (40, 72), the radial forearm flap or adipofascial flaps which can be based on the radial or ulnar (73) perforators, or local muscle flaps utilizing the flexor carpi ulnaris or brachioradialis. However, with burns in this area, these local flaps and their pedicles are often within the zone of injury. We therefore have a low threshold to utilize free flaps, particularly given the plethora of vascular targets and the increased speed at which they can begin rehabilitation (24). Our typical free flaps include the anterolateral thigh flap, parascapular flap or superficial circumflex iliac artery perforator flap (Figure 2).

## 5. Palm

Burn injuries that require grafting or develop contracture tend to be less common due to the thicker, glabrous skin on the palm. Of 603 burned palms at Massachusetts General Hospital, only 17.9% of these required grafting (10). The differing quality of this skin also requires a different set of reconstructive options than previously discussed. Palmar contracture results in flattening and narrowing of the metacarpal arch and difficulty with grasp (74). To re-create this thin, mobile tissue that is adherent to the fascia, skin grafting is often the first choice. However, Pensler et al. (75) showed no difference between revisions or functional results split and full-thickness grafts for the palm after three years, and Al-Qattan (76) showed a decreased risk of contracture with full-thickness grafts over time and that split-thickness grafts were more likely to need repeat grafting. Structurally the skin from the palm fundamentally differs from other skin in terms of its function and appearance. The plantar foot represents the closest match. In a review of split-thickness skin grafting from the plantar arch vs. other sites, Bunyan et al. showed that these grafts were more likely to be a match for color with improved stability and decreased recurrent contracture compared to grafts from the thigh (77).

With exposed deeper structures, the functional priority for palmar reconstruction focuses on restoring tendon glide and restoring adequate soft tissue coverage for vascular and nerve structures. Restoring sensation and contour are also important considerations as both are integral for prehension. Perhaps the ideal flap that best adheres to these functional requirements is the medial plantar artery flap or “instep” flap. It provides a similar color and texture match to the missing tissue with anchoring of skin to the underlying fascia. It can be harvested with the medial plantar nerve to provide sensation. The harvest of this flap is well described, shown to be up to 6 × 3 cm and the defect replaced with a full thickness graft, typically from the groin (78–80). It can also be harvested with the abductor hallucis to reconstruct contracted or fibrotic thenar muscles (81, 82).

Other innervated flap options include the dorsalis pedis flap and the lateral or extended lateral arm flap. For larger defects muscle flaps like the gracilis or latissimus can resurface larger defects and will often thin after de-innervation (83). However with no fascial layer, subsequent fibrosis and scarring may make secondary procedures more difficult (84). Fasciocutaneous flaps like the parascapular and anterior lateral thigh can be easily raised for secondary procedures (8). As in the dorsum, a fascia-only flap followed by skin grafting is also a good alternative including the local reverse radial forearm adipofascial flap or the temporoparietal fascial flap (85). Finally, as with the dorsum, groin or paraumbilical artery perforator flaps can be used although they present more difficulty in terms of positioning and bulk (86).

## 6. Dorsal hand

Burn injuries of the hand often involve the dorsal surfaces; covering the face with the palm of the hand is a natural protective mechanism that results in dorsal hand injury. These injuries may result in dorsal wrist contractures, first webspace adduction contractures, or contractures across the fingers. The latter often results in a claw deformity with the MCP joints held in hyperextension and PIP joints flexed. This pattern of injury is often described using the Graham classification which can aid in determining the depth of injury and likelihood of needing capsular release, in addition to soft resurfacing (87). When considering this area, the goal is to restore the thin, supple, pliable skin in which there is very little subcutaneous fat and through which the contours of the subcutaneous structures are often easily visible.

For superficial defects of the dorsum of the hand and first webspace, skin grafting remains the standard therapy after release of the entire defect. If deeper structures are exposed, flaps are often indicated. In our algorithm, the primary work-horse flap that is easy to harvest and is capable of resurfacing the entirety of the dorsum is the reverse radial forearm flap. If the patient is radially dominant or does not have an intact arch, this can be converted to either an ulnar artery flap or based off of a radial artery perforator in order to avoid sacrifice of the radial artery. Other flaps include the reverse posterior interosseous artery flap (88) and a turnover adipofascial flap (89). Even with very thin local fasciocutaneous flaps there will still be a difference in terms of the contour, color and thickness of the resulting flap which will differ from the original skin (90).

Adipofascial flaps may represent a compromise. Long used for a variety of wounds on the dorsum of the hand and fingers using a 1 or 1.5 ratio for small defects (89, 91), Deal et al. (92) expanded this by using up to 10 × 18 cm flaps from the dorsal forearm in order to resurface large dorsal wounds. The reverse radial forearm can also be taken as a fascial or adipofascial derivative (93). These can be immediately skin grafted to allow for single-procedure, thin, pliable coverage with less donor morbidity than the full fasciocutaneous flap. However as stated, split-thickness skin grafted tissue may remain less durable and depending on the graft take and quality may have a less aesthetic result (90).

The most complex choice of reconstruction free tissue transfer. The key to flap selection is finding a thin, pliable flap that matches the contour of the recipient site. In a review of a multitude of free flaps including muscle, fascial and fasciocutaneous flaps for dorsal hand defects, Parrett et al. (90) showed that fasciocutaneous flaps were the bulkiest and the most likely to require later revision at 67%, with muscle flaps 32%, fascia only flaps 5.8% and finally venous flaps requiring 0% revision. Therefore care should be taken with flap selection to identify a flap that not only covers the defect but also re-creates the “like for like” principle. Consideration should also be given to harvesting in a suprafascial plane or secondarily thinning prior to inset. Typical free fasciocutaneous flaps that would be considered would be the ALT, MSAP, SCIP or lateral arm flaps and typical muscle flaps would be the gracilis or latissimus.

## 7. Fingers

For smaller finger defects, full thickness grafts can be a relatively thin, pliable option. Additionally, local finger flaps (V-Y, cross-finger, reverse cross-finger) can be used. More significant burn injuries to the fingers can result in the burn claw hand which is characterized by the MCPJ in extension and the PIPJ in flexion. Utilizing the classification schema by Graham et al. (87) can be helpful in identifying the depth of injury and need for capsular release. Release of the joint can be performed followed by focal release of deeper scarred tissue or joint capsule. Fufa et al. advocated for wide excision of the scar, freeing all three finger joints and then releasing deeper structures via tenotomy, tenolysis, lateral band mobilization or intrinsic release as necessary through small windows that can be re-closed to re-create a soft tissue plane and allow skin grafting (94). Kirschner wires can be utilized to maintain full extension while the soft tissue heals but must be balanced against the need for early range of motion.

**Figure 3:**
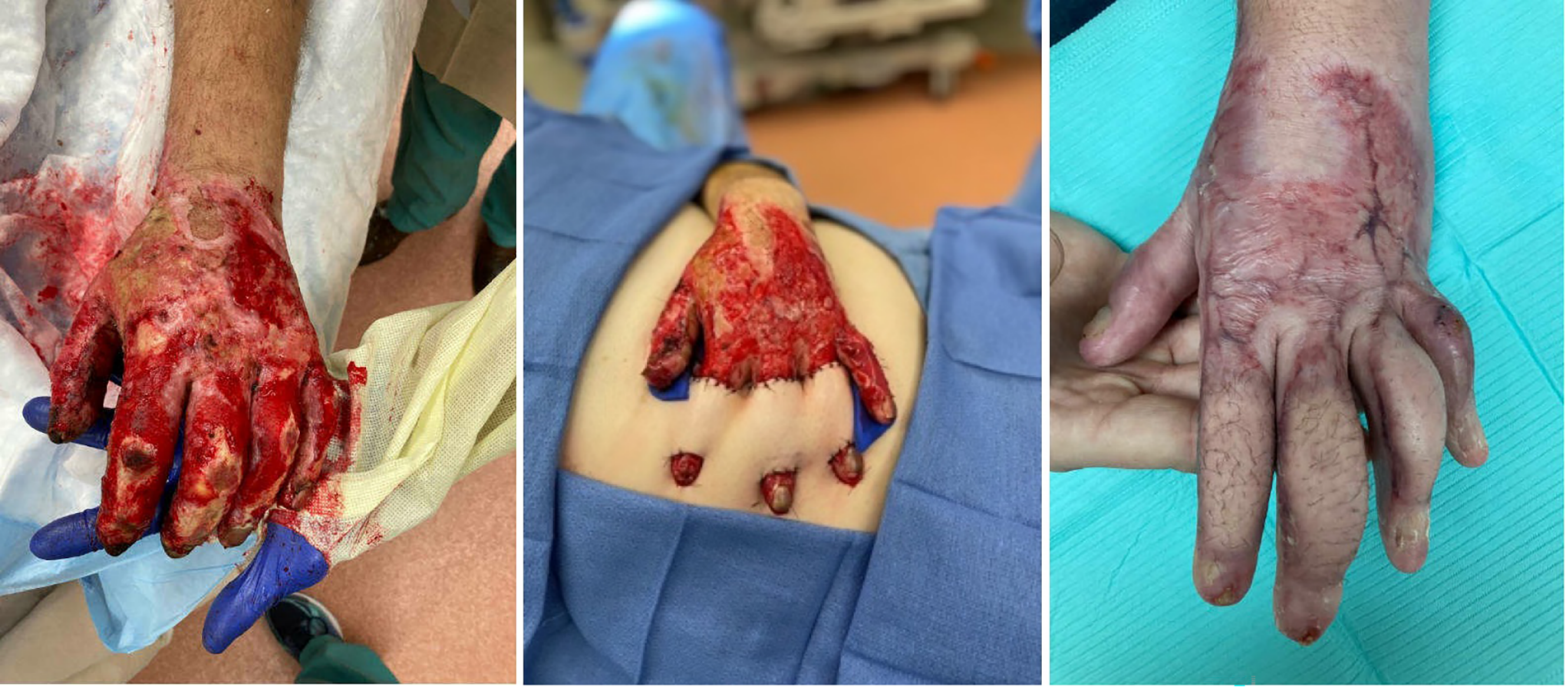
Thirty-five year-old male involved in a motor vehicle collision with subsequent fire and burn injury. Underwent periumbilical flaps for coverage of his dorsal index, long and ring fingers.

For larger defects, numerous local flaps have been described. Quaba et al. (95) initially wrote about using the dorsal metacarpal artery flap for dorsal proximal phalanx and webspace defects and Zhang et al. (96) extended the angiosome of this flap using cutaneous branches in order to cover larger defects. When utilizing this flap, we prefer to maintain a skin bridge at the base of the flap to add venous dermal outflow. Pedicled flaps like the reverse radial forearm and posterior interosseous can be used, potentially syndactylizing the fingers for the initial healing period (97). Finally the abdominal bridge flap can be used in which bipedicled random pattern flaps can be raised to cover individual or multiple dorsal finger defects (Figure 3) (98). As shown by Jabaiti et al. however (49), these smaller, more random pattern flaps may be more prone to failure, especially if lacking a healthy vascularized wound bed.

## 8. First webspace

The webspaces represent the interplay between the soft, pliable dorsal skin and the glaborous skin of the palm. With any reduction in the supple nature of this tissue, motion can be significantly impaired. Release here is one of the most common required procedures in the burned hand (10, 99). Sandzen classified these as mild, moderate or severe based on the magnitude of damage (100). In the first webspace, contraction results in impairment of abduction, adduction, opposition and circumduction, all of the essential movements of the thumb which provide integral function (101). Of these, opposition has been shown to be the most related to improved DASH scores (102). The five essential principles to restoring first webspace function were laid out by Greyson et al. (103) which include providing adequate tissue to re-establish full range of motion, completely releasing the contracture restricting the first carpometacarpal, restoring the width of the palm and the transverse metacarpal arch, releasing leading-edge contractures and restoring normal contour.

If a larger defect is created, this can be replaced with split or full thickness skin graft similar to the replacement of dorsal skin as discussed above. The thumb carpometacarpal joint should next be released in order to allow full circumduction and also replaced with skin grafts if able. Once these primary contractures are addressed then leading edge contractures can be better examined and corrected using local tissue re-arrangement with multiple z-plasties (Figure 3) (103).

Mild contractures in which either the dorsal or palmar surface is undamaged can be lengthened using 4-flap or 5-flap (which incorporates a V-Y advancement) z-plasties, with the 90 degree and 120 degree 4-flap z-plasties shown to gain the most length (104). Though the 4-flap z-plasty theoretically has the most gain, it is also a difficult transposition with the middle flaps requiring significant mobility to achieve their final position, which is particularly true of the 120 degree 4 flap Z-plasty. For more significant contractures in which the CMC joint is restricted, a staged approach should be undertaken which first starts with the release of the contracture with the goal of returning palmar skin volarly. The contracture can be released in parallel without excising additional tissue in order to preserve all tissue present (103).

**Figure 4:**
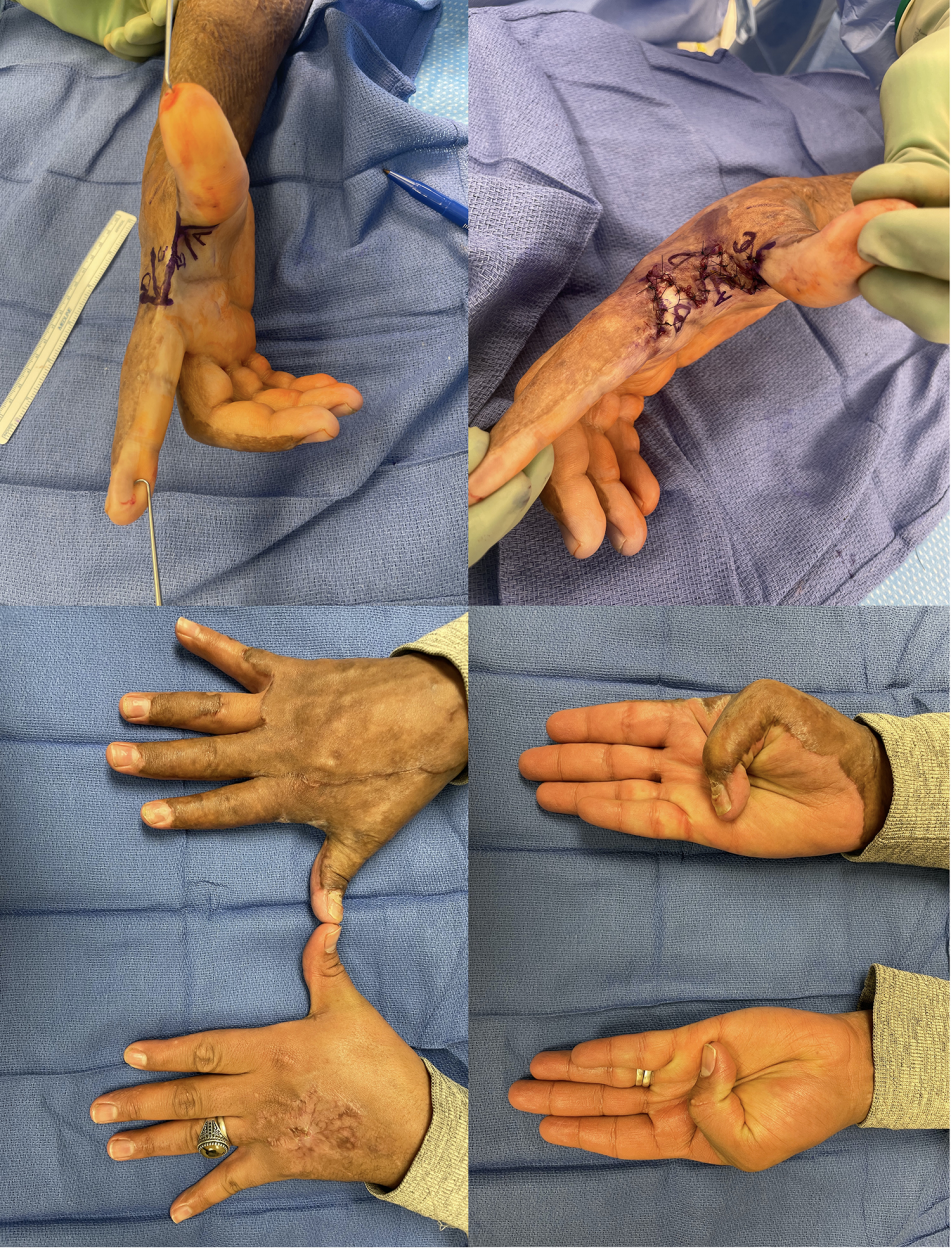
38yo M with a grease burn to his right hand treated initially with STSG. Underwent 4-flap z-plasty for 1st webspace contracture. (**A**) Initial markings, note the release parallel to the contracture. (**B**) Following inset; transposition given the surrounding burn can be difficult (**C,D**) following full release with restored motion.

**Figure 5:**
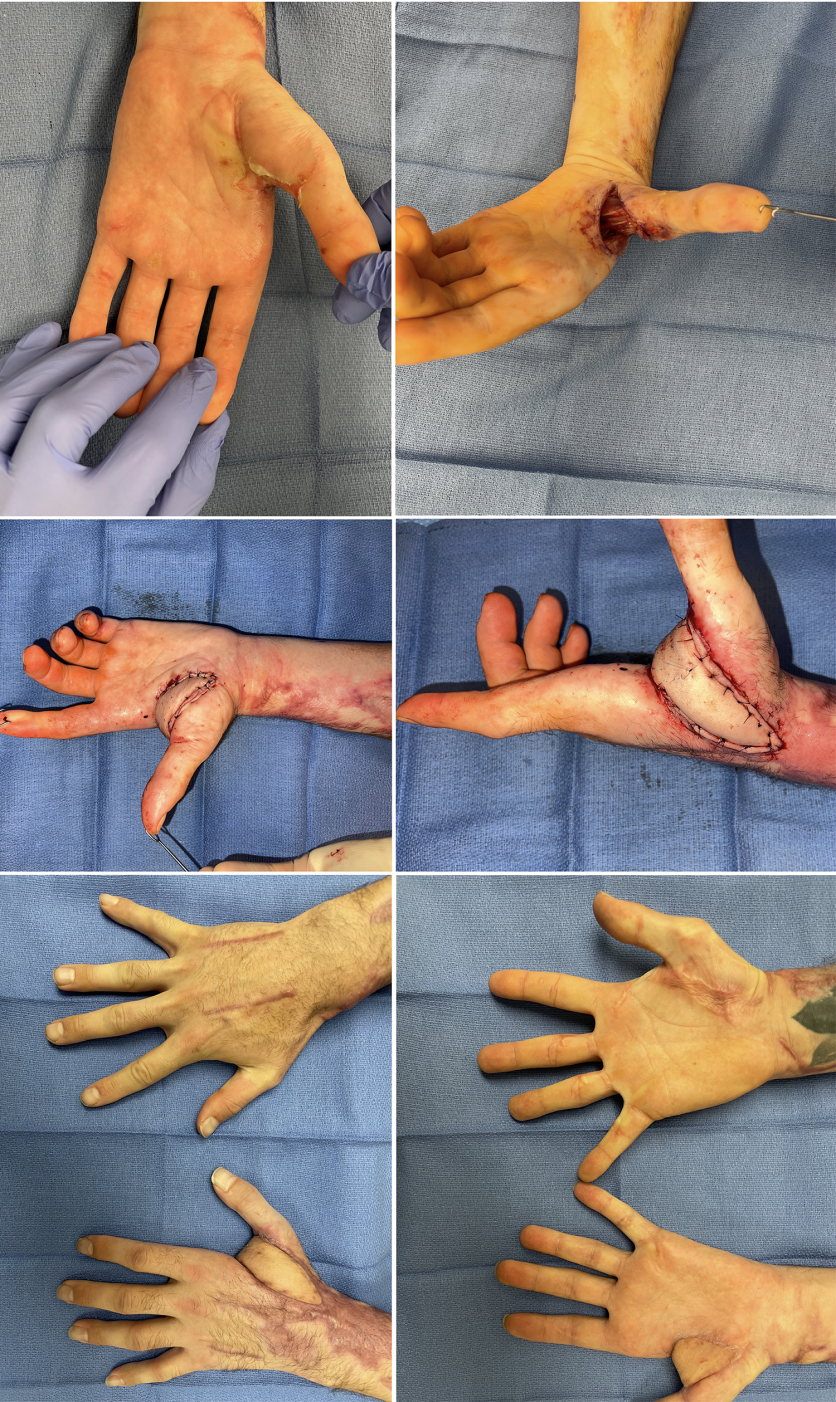
33yo M sustained an electrical injury. Attempted Integra and skin grafting which failed. He also had significant nerve damage to his median and ulnar nerves with high likelihood for additional reconstruction. A free SCIP was performed. (**A**) Initial tight 1st webspace with chronic wound (**B**) after initial release of webspace with exposed structures (**C,D**) SCIP flap with immediate motion (**E,F**) comparable range of motion to contralateral side.

Flaps, either local or free, we reserve for the more complex reconstructive patient. In these patients there is typically a deficit of both volar and dorsal skin, exposed structures that require vascularized tissue coverage or the case is one in which additional reconstructive procedures will need to be performed and access needs to be maintained (Figure 4) (90, 103). Reverse posterior interosseous artery, fascia-only, free lateral arm, and SCIP flaps represent good options in these cases (Figure 5) (105, 106).

## 9. 2–4 webspaces

**Figure 6:**
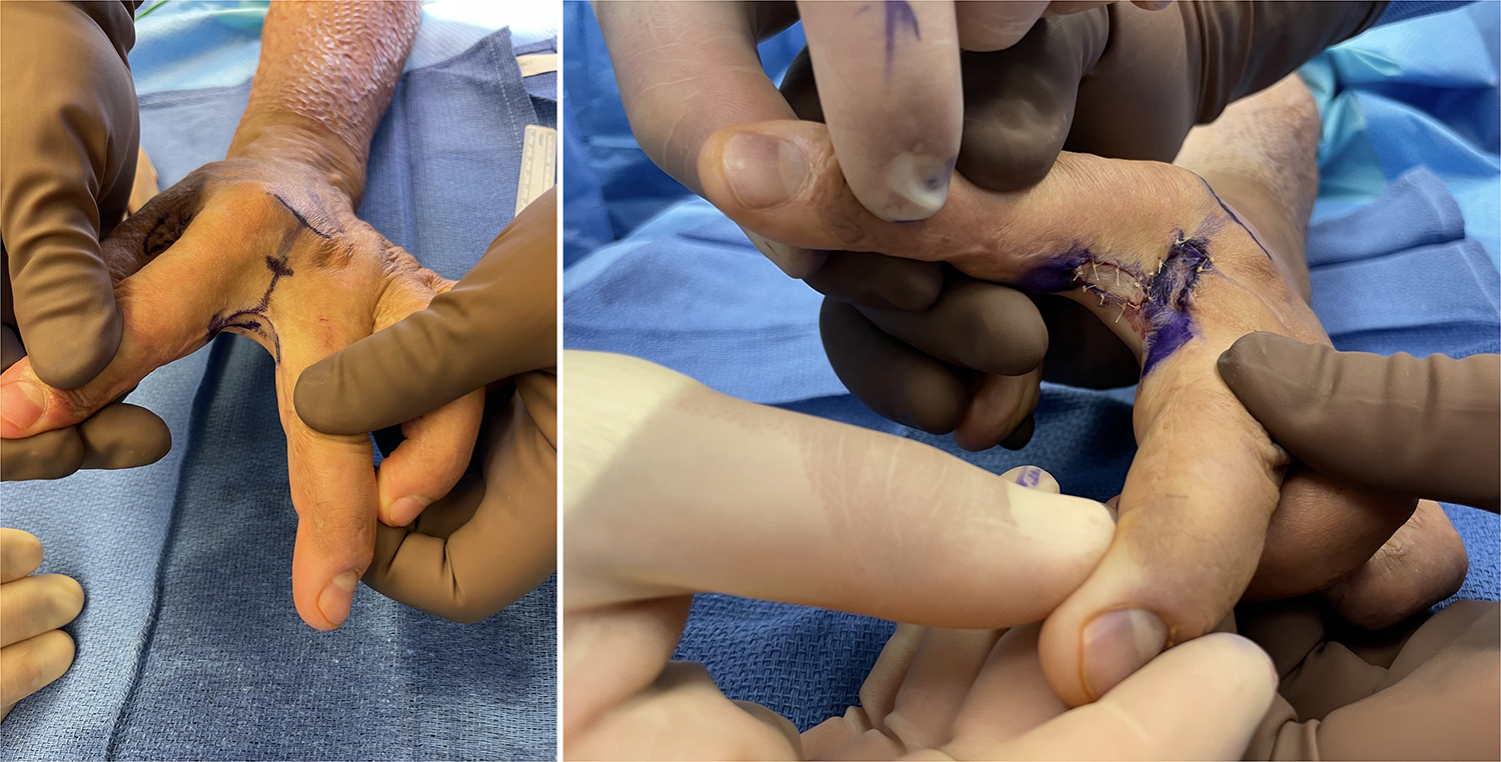
32yo M with 27% TBSA flame burns treated with STSG. He developed 2nd webspace contracture treated with ulnar sided index finger transposition flap and full thickness skin graft to the donor from the antecubital fossa.

Gulgonen et al. (107) classified these into dorsal, palmar or interdigital (syndactyly) type contractures. In dorsal contracture the dorsal surface and interdigital area are both affected, whereas with palmar injuries the interdigital surface is often spared based on the tetrahedron shape that protects the dorsal skin. Therefore especially in dorsal injuries that require a resurfacing of the full web-space, z-plasty does not tend to be satisfactory and can lead to web space creep (108). Instead, fresh tissue must be brought in. A variety of options exist for this including posterior rectangular flaps (108), side of the finger rectangular flaps (107), pentagon (109), hourglass (110) or rhomboid flaps. Similar to interdigital contracture, these can be treated by a similar technique as described for congenital syndactyly (Figure 6).

For palmar defects, full thickness grafts can be placed after complete release of both the palmar and digital contractures. These can be released in a z-shape in order to restore good quality skin between flexion creases (107).

## 10. Adjuvant therapies

Recurrence after burn scar reconstruction is difficult to study based on the individuality of each burn contracture and the various modalities used for reconstruction. Once released however, therapy is critical to maintain the surface area gains. Standard therapies are similar to those in the immediate post-burn recovery phase which include pressure garments, silicone dressings, splinting, and early intervention with physical and occupational therapy.

Laser therapy has arisen as a modality that has been shown to improve the pliability, texture, contracture and pruritic nature of scars (111). Fractional CO2 has been shown in systematic review to have improvement in the thickness and pliability of scars as well as reduction of pruritis and pain (112) and has been recommended by international consensus to be the most effective treatment of scar pliability, thickness and contracture (113). Issler-Fisher et al. (114) also found a reduction in the need for conventional reconstructive therapy by close to 25% after introduction of ablative fractional laser therapy, possibly related to earlier intervention and positive influence on laser therapy on scar maturation. The combination of surgical scar release with local tension relieving soft-tissue re-arrangement and laser therapy, which can increase pliability and improve texture of the surrounding tissue, may create an additive effect, reducing the need for larger surgical interventions (115).

Fat grafting is another procedure used to soften and increase the pliability of scars. Thought to bring in adipose derived regenerative cells thereby increasing vascularity, and increasing collage deposition and remodeling, the limited studies performed have shown improvements in skin texture, thickness and overall satisfaction with the scars (116). In the hand it has been shown to improvement movement and ability to perform activities of daily living (117, 118).

Finally, medical needling represents a burgeoning treatment that has emerged as an alternative, less invasive therapy. Utilizing microneedles that pierce into the dermis, the goal is to stimulate the body's own regenerative system (119), which may induce collagen and improve scar hydration (120), reducing erythema of burn scars (121).

## 11. Conclusion

Contractures can lead to serious functional impairment. Here we present our algorithm and treatment options for each portion of the upper extremity. Reconstruction should proceed with focus on both restoring function as well as aesthetic appearance as best as possible. By following the principles of replacing “like with like” and advancing on the reconstructive elevator, the best reconstructive solution can be found.

## Author's note

The views expressed in this paper are those of the authors and do not reflect the official policy or position of the Department of the Navy, Department of Defense, or the U.S. Government.

## Author contributions

CM: primary authorship, editing and review. MI: editing and review. MG: Origination, editing and review. All authors contributed to the article and approved the submitted version.

## Conflict of interest

The authors declare that the research was conducted in the absence of any commercial or financial relationships that could be construed as a potential conflict of interest.
